# Present but Absent—Nurses’ Experiences of How a Hospital Design with Only Single Bedrooms and the Technological Shift Affect Nursing

**DOI:** 10.3390/nursrep14040196

**Published:** 2024-09-26

**Authors:** Anne Karine Østbye Roos, Vigdis Abrahamsen Grøndahl, Ann Karin Helgesen

**Affiliations:** 1 Intensive Care Department, Østfold Hospital Trust, P.O. Box 300, 1714 Gralum, Norway; 2 Faculty of Health, Welfare and Organisation, Østfold University College, P.O. Box 700, 1757 Halden, Norway; vigdis.a.grondahl@hiof.no (V.A.G.); ann.k.helgesen@hiof.no (A.K.H.)

**Keywords:** nursing, hospitals, single bedrooms, technology, qualitative research, conventional content analysis

## Abstract

Background: Hospitals are complex environments that bring together diverse aspects of technology, society, medicine, and architecture. The aim of this qualitative study was to examine how a hospital design with originally only single bedrooms and technological innovations affect nursing. Methods: Twelve interviews were conducted with registered nurses working in a hospital in Norway. Data were analyzed using conventional content analysis. Results: Results show that a layout with single bedrooms allows nurses to be present and improve patient care, but also complicates patient monitoring, leading to adverse events and patient isolation. Nurses may struggle to find colleagues, experiencing loneliness at work. Additionally, the use of technology, long corridors, and poorly positioned clinical support spaces can contribute to nurses’ mental and physical absence. Conclusions: This study provides knowledge that nurses working in a hospital environment designed with single bedrooms must modify their workflow and communication strategies. Technological advancements are necessary to support nurses’ presence and patient safety, and systems and clinical support spaces must be adequately adapted. Technologically advanced hospitals with only single bedrooms can make nurses feel both present and absent in patient care. This understanding holds significance in practical terms, offering insights to guide future hospital design and nursing practices.

## 1. Introduction

The importance of physical environment and how hospitals should be built were described by Florence Nightingale as early as in 1863 [1]. Nightingale argued that it was most practical to group patients in large wards as this would reduce construction and administrative costs as well as the ease of discipline and oversight. Patient room layout is a debated topic [2] and there is an international tendency to construct hospitals with a larger proportion of single-occupancy rooms [3,4]. Several studies have addressed the effect hospital design has on its users [2,3,4,5,6,7,8,9], and when it comes to operations management, Slack [10] describes how workplace conditions can hinder staff efficiency and organizational productivity. According to Slack [10], organizations engaged in high-visibility operations, such as hospitals, recognize that the appearance of their facilities and processes greatly influences the patient experience. The physical environment of hospitals is, however, often overlooked, although it plays an important role in strengthening productivity and nurses’ performance [8]. Research indicates that nurses’ experiences of working in hospitals featuring single bedrooms can differ [2,3,4,9]. Some nurses appreciate the privacy and focus that is offered by single bedrooms as it allows them to provide more personalized care and communicate better with the patients [11,12,13]. Others find it challenging to monitor patients in single bedrooms, leading to concerns about patient isolation and safety [3,14,15,16], as well as increased walking distances [13].

Healthcare facilities are intricate structures that integrate various elements of technology, society, medicine, and architecture [2], but according to Booth [17], there is a reluctance to adopt digital technology in healthcare. In the digital age, nurses must embrace digital practices, which may challenge traditional perspectives on the nursing role [17]. While the incorporation of technology in nursing practice presents challenges, the benefits of improved patient care and enhanced healthcare delivery make the endeavor rewarding [18]. Booth describes that technology can be perceived as a distraction or also as an unwelcome interference in the hands-on caregiving role and the relationship that nurses form with patients and their next of kin [17]. Involving nurses in developing digital solutions is therefore recommended [19].

Given the implementation of hospitals predominantly featuring single bedrooms and an augmented array of technological systems, it is imperative to examine nurses’ perceptions of these innovations and how it affects their work. Despite the extensive research on nurses’ experiences of working in different hospital settings, there remains a significant gap in understanding how the design of single-bedroom and technological innovations affects nurses. This study aims to address this gap by investigating nurses’ experiences, using qualitative interviews to uncover insights that have been previously overlooked. By addressing this gap, the study aims to enhance knowledge about how hospital design affects outcomes, potentially informing future hospital planning and policy development.

Traditionally, nurses recorded patient care using pen and paper. Technological innovations and digital transformation have led to a shift from paper to computer-based documentation and the use of software and devices designed especially for nurses. In this study, the term “technological innovations” refers to the use and integration of sophisticated technologies and digital solutions within nursing activities. This includes among others, electronic health records, different mobile devices with different applications, medication management systems, support systems, and sensors. These systems help nurses record, store, and transfer the patients’ health and medical information.

## 2. Materials and Method Design

This study had a descriptive design with individual semi-structured interviews. The manuscript adheres to the COREQ (COnsolidated criteria for REporting Qualitative research) Checklist [20].

### 2.1. Study Setting

The research was conducted within a hospital trust located in the southeastern region of Norway. Serving a population of approximately 320,000 individuals, the hospital is considered medium-sized by Norwegian standards [21].

**Figure 1:**
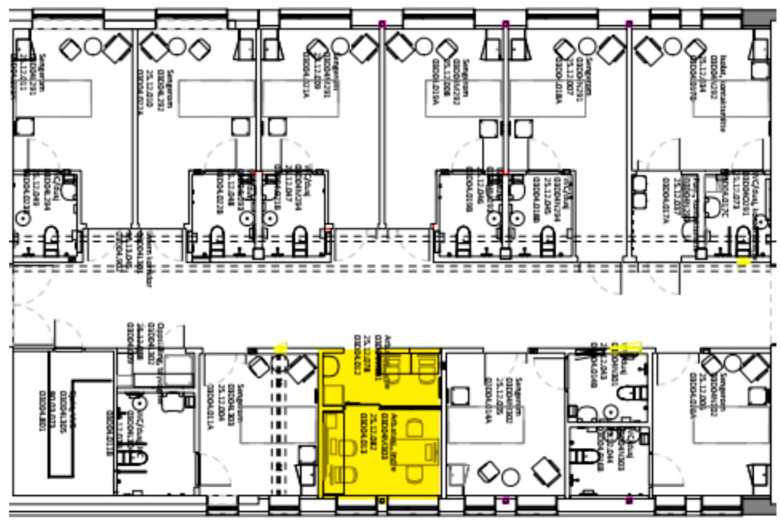
Bed court with 9 beds and one nurse station (highlighted) [22].

**Figure 2:**
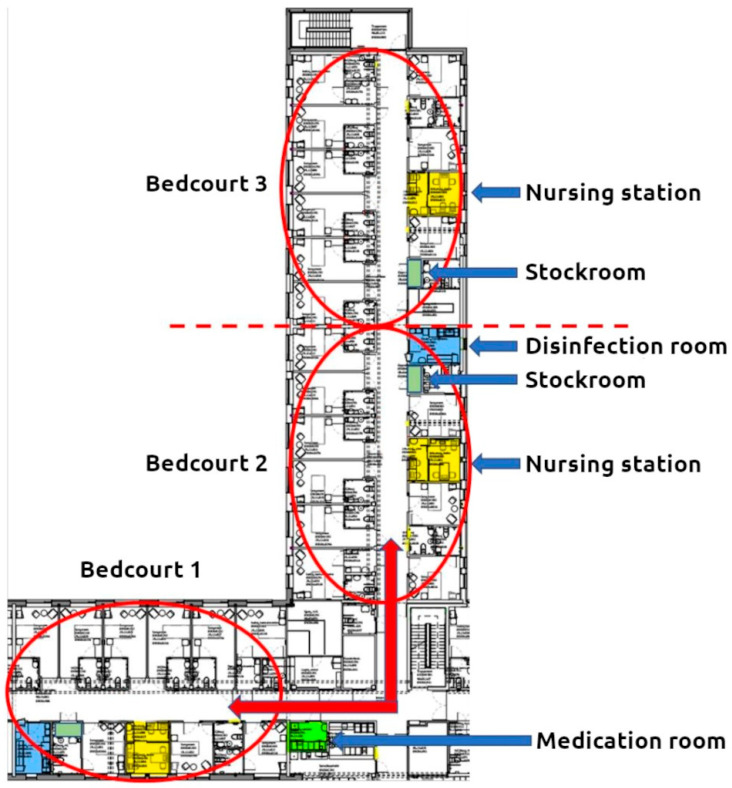
L-shaped ward consisting of 3 bed courts (marked with a red circle), with nursing stations (highlighted), stockrooms, disinfection room, and medication room [23].

The hospital was originally constructed with 257 single bedrooms and private bathrooms; however, space constraints and a lack of beds led to the conversion of 47 of the single bedrooms into twin rooms. Each ward featured a dining room with a buffet but lacked additional dayrooms or gathering areas for patients. Wards comprised three or four bed courts, each equipped with a small, decentralized nursing station containing a limited number of computers and workspace (Figure 1). Attending physicians utilized the computers and workspace within the nursing stations when present. The hospital layout included long corridors with some wards adopting an L-shaped design (Figure 2). Design choices included small stockrooms within the bed courts and a larger stockroom located away from the patient area.

The hospital was built in 2015 and has sophisticated information and communication technology (ICT) system solutions. Most of the systems were implemented when the hospital was new and HIMSS (Health Information and Management Systems Society), an international organization assessing hospitals’ adoption of modern ICT system solutions, has ranked the hospital at level six out of seven in their evaluation framework [23]. The different electronic systems were used across various operational procedures to aid both healthcare and supporting staff in their duties. Multiple technology devices were configured for use in patient rooms, hallways, and nursing stations enabling nurses to work with greater flexibility. Systems included, among others, electronic patient records, electronic charts, “closed-loop” systems for electronic medication administration, blood transfusions, blood tests, and breast milk administration. Staff undergo training in the utilization of various systems as part of their onboarding process.

### 2.2. Nursing Workflows

In the hospital where this study took place, nursing personnel operated in teams assigned to each bed court. Each team included registered nurses holding bachelor’s degree and healthcare assistants. They followed established protocols to maintain a standardized workflow that was customized to meet the specific care plans and nursing requirements of each patient. The integration of advanced ICT solutions within the hospital will most likely impact nursing workflows, particularly in processes such as patient admission, documentation, care planning, vital sign monitoring, medication administration, and discharge planning.

### 2.3. Entrance to the Field and Sample

Permission to conduct the research was sought from the heads of the medical and surgical departments, as well as the research department in the hospital. Participants were subsequently recruited through their nurse managers. They received written information about the study and written consent was obtained. Inclusion criteria for the study required participants to be registered nurses working in either surgical or medical wards, willing and able to articulate their perspectives on the topic, and experienced in caring for patients in both multiple and single bedrooms. Nurses meeting these criteria were instructed to contact the third author for an interview.

### 2.4. Participants

A total of 15 nurses reached out to the third author, of which the first 12 were included in the study. After having interviewed 12 nurses, no new information emerged and the information strength was strong. The age of participants ranged from 25 to 53 years, with a median age of 33.5 years. Their nursing experience spanned from three to 30 years, with a median of 10.5 years. Only one nurse had a specialization in nursing. Prior to the hospital’s renovation, which converted some patient rooms into double occupancy, all nurses were accustomed to working in single-patient settings. Despite their varied departmental assignments, the nurses collectively provided care that involved a moderate level of fundamental nursing services. See Table 1 for an overview of the participants’ characteristics.

### 2.5. Data Collection

The data were collected through private interviews that were conducted between fall 2022 and early winter 2023, all by the third author. A semi-structured interview guide was used with open-ended questions like: “What kind of advantages or disadvantages do you think the hospital structure with single bedrooms represents?” “Can you share your experiences with the different technical programs that are being used in the hospital?” “In what ways do you feel that the wards are designed to ensure interaction with the patients?” Follow-up questions were asked to have the nurses elaborate their answers. The interviews took place privately over the telephone, and lasted between 29 and 47 min, with a medium range of 40 min. Some of the interviews were disrupted due to technical difficulties but were continued within minutes. The interviews were recorded and transcribed accurately by an external transcriber who had signed a non-disclosure agreement.

### 2.6. Data Analyses

Data were analyzed by using conventional content analysis as described by Hsieh and Shannon [24]. Initially, the authors thoroughly examined the transcript multiple times to make sure data were saturated, and to gain a comprehensive understanding and perspective of the material. The first author proceeded to analyze the text meticulously, reading it word by word. Important sentences and words that appeared to capture the key thoughts of the nurses were identified and marked. Codes and a number of categories were then assigned directly from the transcript and initial reflections were written down along with preliminary analysis. The codes and categories were sorted, discussed, and modified into a smaller set of categories by all authors until consensus was reached (Table 2 and Table 3). Excerpts from the material were selected to strengthen the trustworthiness of the results and illuminate the different categories.

### 2.7. Ethical Considerations

The study followed the principles of the Declaration of Helsinki [25] as well as the guidelines for nursing research from the Northern Nurses Federation [26]. The study was endorsed by the Norwegian Social Science Data Services (by SIKT) (Number 803687).

The participants were given written information about the study and about the researchers, and a written approval to participate was obtained. Participants were also informed that they could withdraw from the study at all times without any consequences. The participants’ identity is only known by the third author. Data were anonymized and administered confidentially throughout the study and audio recordings were deleted after they were analyzed.

## 3. Results

Two categories with three subcategories each were identified from the data, describing nurses’ experiences of how a hospital design with only single bedrooms and technological innovations affect nursing (see Table 4).

### 3.1. Being Present

In the interviews, nurses gave examples that the hospitals’ architectural design with single bedrooms allowed them to be present both physically and mentally when performing their tasks. A setting with single bedrooms was perceived by the nurses to enhance the interaction according to the individual patient’s needs. This was described by the nurses to increase their awareness and their sense of being able to connect with the patients. Being present contained the subcategories “the enhanced communication”, ” the placement of patients”, and “the quality of nursing”.

#### 3.1.1. The Enhanced Communication

The data showed that nurses found it easier to communicate with patients in single bedrooms as they were able to be present both physically and mentally when interacting with the individual patient. The nurses claimed that this setting allowed for deep conversations where nurses and patients could speak more freely. Our findings indicate that the design featuring single bedrooms allowed for increased nurse presence, which in turn enhanced the provision of emotional and psychological support. The nurses valued this, especially if they were to ask patients personal questions or talk about serious matters like handling a life-changing diagnosis. Most patients seemed to be more willing to share information when staying in a single bedroom as one nurse declared:

*“If it’s questions about life and death, or drug problems at home, children… it is much easier to address these things and talk openly about them.”*

Nurses stated that both staff and patients seemed to withhold information when in twin rooms. Patients did not disclose things they thought were embarrassing, and staff held back information to protect the patients’ privacy.

The nurses claimed that a design with single bedrooms allowed them to adapt the conversation to the patients’ needs and offered a better environment for interacting. As well as enhancing communication, nurses found it easier to maintain confidentiality in single bedrooms and mentioned this as the setting’s main advantage. Nurses stated that breaching confidentiality was a concern for them and something that happened on a regular basis in twin rooms. The data indicate that the presence of nurses with patients facilitated the sharing of confidential information. The nurses believed that this information may not have been disclosed had the settings not been structured to promote such engagement. However, nurses described that some patients did not seem to be concerned if others were listening in during conversations. One nurse exclaimed:

*“The patients can say, nah…I have already informed my co-patient about this, so I don’t mind.”*

Overall, data showed that the nurses described that a confidential environment of a single bedroom where they were able to be present both physically and mentally enhanced the communication with the patients.

#### 3.1.2. The Placement of Patients

The data suggested that conscientious placing of patients significantly added to the nurses’ presence as nurses described that the patient’s individual need for supervision guided their placement in the ward. Patients were placed close to the nurses’ station for safety reasons if they had mental sufferings, agitation, anxiety, were clinically unstable, or had a risk of falling or climbing out of bed. Nurses also placed patients not capable of using the alarm system in close proximity. Strategically placing high-risk patients near the nurses’ station was said to facilitate quick responses during emergencies, thereby promoting a safe environment in which the nurses could be present when critical.

Keeping the bedroom doors open was a common measure to hear if patients were yelling for help, and a means to tend to patients’ increased need for care and attention. This simple act made nurses feel present as one nurse stated:

*“We cannot observe the patient behind closed doors.”*

Several nurses claimed that the units’ layout prevented them from observing the patients as much as they wanted. This was clearly a stressful factor, leaving nurses with the feeling of not tending to the patients as much as necessary. One nurse shared that she had found patients that had suffered a cardiac arrest alone in their rooms. Other nurses claimed that patients could be deteriorating without staff noticing. Nurses therefore saw the value in placing patients in multiple bedrooms. The nurses were concerned about the number of fall incidents, although some of the nurses stated that the number of falls had decreased. One nurse commented:

*“It happens all the time that patients are falling in the bathrooms. It’s impossible to see them and the rooms are soundproof, and the doors are closed, so you cannot hear them yell either.”*

Nurses with experience of working in multiple bedrooms told that co-patients played an important role in fall prevention or alerting the nurses if the co-patient was in need:

*“Some patients take on the responsibility to speak up on behalf of others. After all, there is no one that speaks up for patients in solitary confinement if they are not capable of doing it themselves.”*

#### 3.1.3. The Quality of Nursing

According to the nurses, single bedrooms made it easier for them to perform person-centered care and to focus on the tasks at hand as they were able to be present mentally and physically. Nurses claimed that single bedrooms added to an increase in the quality of care because the rooms were spacious, ergonomically adapted, and had room for equipment. The converted single bedrooms did not add to patient safety as one nurse stated:

*“Patient safety in single bedrooms is so much better, just because there is lack of space in the twin rooms…”*

The lack of space was an issue when handling emergencies, and when patients required moving assistance or needed help with personal hygiene. Nurses also described that fall incidents were common in the converted single rooms due to the doubling up of miscellaneous apparatus, which made it more difficult for patients to move around.

From an infection control perspective, the nurses expressed that single bedrooms offered a better environment and that it was easier to prevent contagious diseases. Another important benefit afforded by single bedrooms was medication safety as one nurse exclaimed:

*“Considering the medication distribution... it’s not so easy to make mistakes. Mixing patients up etcetera, so in that sense it is probably better to have a private room. I think so.”*

Overall, the nurses in our study expressed that single bedrooms improved the quality of nursing, as they experienced fewer disruptions while carrying out their responsibilities, enabling them to focus more effectively on their tasks. However, several of the nurses stated that they would have preferred a hospital with a combination of single and double bedrooms. From their point of view, this would have catered to both the nurses’ and the patients’ needs.

### 3.2. Being Absent

The data showed that the hospital’s architectural design and technological equipment also left nurses feeling absent from the patients. The subcategories “the encumbrance of technology”, “the time-consuming layout”, and “the lonely patient” describe the nurses’ reflections on absenteeism.

#### 3.2.1. The Encumbrance of Technology

In our material, nurses described that the decentralized workstations were too few and too small and made it difficult to be present for the patients. Nurses claimed that the attending physicians often occupied the workstations and the computers leaving the nurses with nowhere to sit:

*“We get degraded when the doctors are coming, and then we are left to use the portable computers. We have to stand and document out in the corridors.”*

Nurses described a disconnection between the number of computers available and the hospital management’s expectations for them to embrace the new technology. Most of the nurses exclaimed that they had received sufficient training in using the different systems. However, several stated that the technology did not meet their needs as the different systems or programs did not communicate. Problems with hang ups and having to document the same information in as much as four different systems were described. The log in/log out processes of different programs several times a day were explained as time-consuming and stealing time they rather could have spent together with the patients.

The available systems were believed to benefit patient safety, but nurses expressed the systems to be a serious safety concern as the systems sometimes mixed up patients. Several nurses raised concern about the electronic medication management system causing an extra workload for them when administrating medications. Because the systems were too time-consuming to handle, they were not always used as intended. When asked about this, nurses acknowledged and exclaimed that this could affect patient safety.

On the other hand, most of the nurses valued the possibility the different systems offered and did not want to go back to previous schemes. With the introduction of new technology, nurses believed their role and focus had changed. When asked how the technology had impacted their work, one nurse stated:

*“It’s not enough just to be a good nurse at this hospital. There are so many systems that you have to master in order to work here.”*

The nurses did not fully rely on the different systems for measuring vitals and used their clinical gaze as well. One of the more seasoned nurses stated that she did not trust “*those machines blindly*” as it did not always offer compliance between the patients’ clinical state and the given measurements.

Some nurses claimed that many of the systems were not user friendly, and that technological devices and the demand to use them sometimes took time away from the patients:

*“I believe that much more time is spent on administration, documentation and looking at the computer than before.”*

The nurses had received complaints from patients and their next of kin because they were absent and spent too much time on the computer or on the phone. It was also mentioned that some of their coworkers seemed to be caught up in the computer and the technology instead of tending to the patients:

*“If you must sit and read for an hour to get to know the patients, there is something wrong. You have to see to the patients. You have to use your eyes.”*

#### 3.2.2. The Time-Consuming Layout

Nurses described the hospital layout as extremely time-consuming compared to other more conventional layouts. The long walking distances were mentioned by all nurses as a hinderance to being present for the patients, and one nurse stated:

*“The very long distances make it difficult if you’re in a patient room and need to go and get something. If you need bandages for instance, you must walk a long way to the small stockroom and then there might not be any bandages there and then you have to walk further down to the main stockroom, and it is 100 meters away.”*

The nurses mentioned how the bed court’s layout removed time from being with the patients and told that they spent a lot of time just walking back and forth to each room, opening doors and checking. The layout with only single bedrooms was not considered time beneficent nor effective:

*“It takes significantly longer to see to patients in four different rooms, instead of going into one room and tending to all four at the same time.”*

The nurses claimed that the most ideal layout would have been to have the workstation and stockroom in the middle of the corridor with patient rooms centered around. Because of certain design features in the wards, nurses remarked that it was challenging to monitor activities effectively.

The nurses pointed out that they sometimes did not know who was working in the same ward as them before the end of the shift or before they met colleagues in the locker rooms. Not being able to locate colleagues was a concern and nurses exclaimed that a lot of time was spent trying to find someone that could help when needed. Many described a feeling of not always feeling safe because coworkers were not always available or in sight. This was specifically mentioned as being an issue during night shifts or in emergency situations.

Nurses reported that they sometimes felt they were coming up short because of the single-bedroom layout:

*“Sometimes you feel like you cannot leave the patient, but then you have another patient in another room that is just as ill. And you should have been there too.”*

The nurses solved this by trying to prioritize and telling the auxiliary nurses or assistants what to observe and what to do. This could sometimes cause stress and a feeling of inadequacy for the nurses when they could hardly see to all the patients and had to rely on others’ observations.

#### 3.2.3. The Lonely Patient

An issue raised by nurses in our material was that receiving less indirect supervision and therefore less presence of the nurses could affect the patients’ mental health and well-being. According to the nurses, single bedrooms added to patients’ feelings of being lonely and isolated because they received less of the indirect supervision they would get in a multiple bedroom. Nurses reported concern of not being able to tend to the patients’ needs for human contact as they sometimes only saw to the patient during mealtimes, or when administrating medications or doing ward rounds with the attending physician. When patients were alone and behind closed doors, nurses described that they could not prevent patients from feeling lonely. One nurse stated:

*“We don’t have the time or possibility to sit with the patient for a long time.”*

Another exclaimed:

*“They are at the mercy of the nurse.”*

All the nurses described the lack of social arenas in the wards to be a negative factor for the patients. The word “sad” was used to describe the non-existent common rooms and that patients were left to wander corridors should they wish to socialize. Nurses said that patients frequently asked for a place where they could gather with their next of kin or other patients.

Peers transferring information were considered valuable, and nurses mentioned it was significant that patients could benefit from each other’s experiences. Elderly bedridden patients were not able to participate in such encounters, and nurses described that a lack of contact with others affected their mental health:

*“They become more dark-minded; they lose contact with the outside world. They feel lonelier and the sense of social exclusion becomes stronger.”*

Nurses expressed strongly that patients could benefit each other’s well-being and told that many patients would use the alarm system just to socialize with the nurses. This added to the nurses’ workload as one nurse pointed out:

*“It would have been nice if they could entertain themselves a little, because it requires a lot from the staff to have to talk with these lonely souls who lie in a private room and see no one but us.”*

The conversion from a single bedroom to a double twin room was mentioned by one nurse as valuable for those feeling lonely:

*“If you get a roommate, at least you will get some more social interaction and see another person.”*

Nurses also exclaimed that the hospital’s design did not always support treatment principles for specific patient groups, especially for those in need of physical activity after surgery. In our material, we found that nurses described that a lack of social arenas and having a private television in every room tended to promote inactivity among many patients.

## 4. Discussion

This study demonstrates how a hospital design with only single bedrooms and technological innovations affect nursing. While the incorporation of single-room accommodations and new technologies facilitated nurses’ presence and engagement with patients, it also resulted in nurses being absent both physically and mentally.

In this study, nurses conveyed that being physically present and attending to the specific needs of individual patients were more manageable in single bedrooms. Care provision in such settings resulted in reduced interruptions [27] and enhanced concentration for nurses [12]. This has been reported to be especially important during the administration of medications as distractions during medication administration have been linked to medication errors [28]. Nurses participating in our study expressed a preference for administering medications in single-occupancy rooms, as it decreased the likelihood of patient mix-ups and subsequent adverse events. Nurses noted a decline in the quality of care and patient safety when dealing with two patients in a converted room. Conversely, nurses expressed that a configuration with only one patient in a single bedroom enhanced the quality of care by augmenting nurses’ presence and enabling them to concentrate on addressing patients’ individual needs. Nurses in this study claimed that single bedrooms afford them the chance to engage closely with patients and establish meaningful connections. Due to the absence of social arenas, nurses in this study noted that most patients spent their time within their individual bedrooms. Consequently, as these bedrooms served as the primary setting for patient–nurse interactions, they functioned dually as care and work environments [7]. Hence, it is significant that the spatial layout of the bedrooms not only enables the effective performance of caregiving duties but also promotes a supportive and therapeutic atmosphere that encourages presence. Presence is crucial for delivering patient-centered care [12,29].

Despite the reduced distractions in single bedrooms, the results indicate that technological devices and systems significantly constrained nurses, who viewed these tools as an encumbrance and potentially detracting from their focus and time with patients. According to Booth et al. [17], there is a persistent issue concerning nurses’ ability to keep up with the rapid advancements in digital technology. When the existing technological infrastructure lacks proper adaptation or resources, nurses find it challenging to fully exploit the opportunities offered by various systems. Benner et al. [30], highlighting the potential overvaluation of technical skills compared to nursing practice, emphasizes the significance of adopting a critical perspective on technology. In the digital era, nurses must reconsider their approaches to patient interaction and care [17]. Nevertheless, the nurses in our study expressed skepticism regarding the notion that technical devices could replace the natural value of the nurse–patient encounter.

In addition to clinical expertise and the capability to assess the outcomes facilitated by technology, it is crucial to analyze its validity [30]. Our findings indicate that being a proficient nurse is not enough; one must also possess digital competencies to effectively manage daily tasks. The integration of emerging technology in nursing is and will continue to be pivotal [17,31]. Our research reveals that time-consuming technological processes impede nurses’ availability for direct patient care and act as a barrier to their presence. This unintended consequence may reshape nurses’ interactions with patients. Technology can be perceived as intrusive and disrupt traditional care practices [17], which may explain why some nurses in our study were hesitant to embrace digital approaches.

In addition to a lack of user friendly technology, inadequate workspace had adverse effects on nurses. Nurses reported instances where they were compelled to yield the use of computers at nursing stations to attending physicians. When constrained to utilizing portable devices in corridors, nurses experienced a sense of degradation. Research has demonstrated that an unsupportive physical environment can have a negative effect on nurses’ well-being [32] and cause stress [2], while overcrowded workstations hinder nurses’ ability to fulfill note-taking responsibilities [4]. The work environment plays a significant role in shaping staff attitudes, motivation, and behavior, potentially fostering or hindering desired behaviors. Such challenges can impede staff effectiveness and organizational performance [10].

Ensuring efficient organizational flow requires building layouts that support ongoing operations, as they significantly influence both effectiveness and resource allocation [10]. Our study demonstrates that the physical placement of stockrooms within the ward has a negative impact on nurses, particularly when they need to retrieve supplies. Strategic and proper placement of stockrooms and nurses’ stations is vital to ensure the efficient delivery of patient care [5,33]. Increased walking distances detract from time being present with the patients, and the distant positioning of necessary equipment affects the utilization of nursing resources. Cusack et al. [34] characterizes this as time wastage for nurses, while Søndergaard et al. [13] notes that nurses experience heightened stress levels due to the extended distances they need to walk and at the same time being absent from patients. Our results indicate that patients will receive less direct care and nurses will be less present if storage rooms and nursing stations are not strategically positioned, and the ward layout does not promote patient visibility.

The significance of patient monitoring was emphasized by Florence Nightingale as early as 1863, advocating for hospitals to be designed in a manner that allowed nurses to oversee patients directly [1]. In our study, nurses perceived the wards’ layout to be lacking in its ability to effectively monitor patients. Nurses mentioned that they attempted to mitigate this issue by allocating patients within the wards according to risk assessment, with a priority on ensuring visibility for the nursing staff. As a result, patient placement within the wards was guided by the need to observe patients, prevent adverse events, and provide necessary assistance. Nurses assigning patients so that they are visible to the nursing staff is a measure to improve observation of patients at risk of deterioration [9,11,28,34,35]

Although nurses in our study reported that they thoroughly controlled patient placements, adverse events still occurred. The nurses told of adverse events where patients had been found unattended in their rooms after experiencing cardiac arrests or falls. Reduced direct observation may contribute to increased falls and other serious incidents going unnoticed [3,7,11,34]. Many nurses in our study expressed concerns about potentially missing critical observations due to their absence and inability to visually monitor patients. This identified concern aligns with findings from other studies [3,6,9,13,27,34]. The layout of the ward can therefore impact patient safety as well as add to concerns for healthcare professionals. When discussing patient safety and the importance of nurses’ presence and ability to monitor patients, it is essential to pay attention to how nurses can attend to patients who are not assigned near the nursing station because they are perceived as low-risk or less demanding. Such patients may not receive the necessary attention or care as they are at risk of being monitored less. Understanding the diverse needs related to the physical environment is therefore important [5,11,36].

Another issue that was raised by the nurses, was the problem of locating colleagues in the unit when needing assistance. This sense of isolation from peers has been termed the “lonely practice” [13] and not having visual contact with colleagues is described as negative to teamwork [11] and learning [9]. Donetto et al. [11] states that a ward design featuring all single bedrooms can create a work environment that discourages collaboration and support among colleagues. The absence of a supportive and collaborative atmosphere can significantly impact nurses, impeding their access to mentorship and guidance, compromising their safety, and potentially leading to situations where poor practices go unnoticed [34].

Aside from nurses occasionally experiencing loneliness in an environment comprised solely of single bedrooms, numerous studies have examined the feelings of solitude and loneliness reported by patients in such a layout [3,14,15,16,37]. Nurses in our study claim that a layout featuring single bedrooms can notably impact the mental well-being of especially elderly patients, leaving them with feelings of social exclusion. When doors are closed, patients are physically isolated from the hospital environment, staff, and fellow patients [13]. This contradicts the fundamental need of patients to feel acknowledged and connected [12,36]. Patients in single bedrooms can feel an unmet need for companionship [13], as well as a sense of an empty and lonely room [15]. Nurses in our study reported that patients became dark-minded when they were unable to socialize and expressed concern about the absence of social arenas for patients to gather. The nurses acknowledged the significance of patients being able to interact with each other, yet with no designated dayrooms or natural meeting places available, patients were left to wander the corridors. This parallels the results in a study by Anåker et al. [15], who found that hospital corridors lack an engaging environment for patient interaction. Nurses in our study stated that the layout featuring single bedrooms did not encourage physical activity. According to Søndergaard et al. [13], a hospital layout with single bedrooms can lead to self-imposed isolation. Patients often remain in bed for extended periods, finding a sense of belonging and comfort in their beds [7]. When social arenas are lacking, our results indicate challenges in mobilizing patients out of bed post-surgery. Nurses expressed concerns about the potential negative impact on patients’ health from this issue. These results suggest that hospital design can significantly influence patient activity levels in a rehabilitation setting. When patients are unable to leave their rooms, they rely on nurses to fulfill their social needs [37]. This demonstrates the importance of facilitating social contact and social arenas for patients who are susceptible to feelings of loneliness. This is easier and more feasible in wards with dayrooms available [37] and where patients can physically see the staff, even if the staff are not caring for them [11]. Therefore, hospitals should be organized and designed to encourage communication between both nurses and patients, with a configuration that enables nurses to be readily accessible and visible.

### Strengths and Limitations

The data in this study were collected from a limited number of nurses and the inclusion of more nurses may have provided additional data. A strength of the study is, however, that the nurses included in the study varied in age and years of experience and that they worked in different wards. This, in addition to the nurses being informative and willing to share their experience, added to rich and diverse data that gave valuable insights on the topic. Interviews were conducted until no new information emerged. Rigor was ensured through maintaining a systematic approach throughout the study. To enhance credibility and trustworthiness, all authors were actively engaged in developing categories, the discussion, and the validation of the results. As the first author is affiliated with the hospital where the study took place, the third author (who is not affiliated) performed all the interviews. This was done to ensure reflexivity so that no potential researcher–participant relationship should influence the research process or the outcome.

This study was conducted in a Norwegian context, and our results align with results from other studies in similar contexts. We have aimed to improve the transferability of our results by providing detailed descriptions of the setting, how the nurses were recruited, and how data were collected and analyzed. Results from this study can therefore be applied to other contexts with comparable healthcare systems.

## 5. Conclusions

This study examines nurses’ experiences in a technologically advanced hospital featuring single bedrooms. The results reveal that such a hospital setup can make nurses feel both present and absent in patient care. This understanding holds significance in practical terms, offering insights to guide future hospital design and nursing practices.

In a hospital with high technological integration and exclusively single bedrooms, nurses state they have the opportunity for increased physical presence and can allocate their attention to one patient at a time. Nurses observe that communication is improved with patients in single bedrooms but mention that a lack of social arenas affects the patients negatively and have concerns in regard to patient isolation and their mental health.

When patients are assigned to single bedrooms, nurses stress the difficulty of adequately monitoring all patients under their care, leading to adverse events and patients feeling lonely due to their limited presence. The use of technological devices, long corridors, and inadequately positioned stockrooms and nursing stations may also contribute to a condition of mental and physical absence among the nursing staff. Nurses may also experience problems with locating colleagues, leaving them feeling lonely at work and with reduced social interaction.

Within a hospital environment with single bedrooms, nurses must modify their workflows and communication strategies to sustain standards of care and patient welfare. Nurses must acknowledge the significance and challenges of technology. Technological advancements are necessary to support nursing presence, and systems and workspace must be adequately adapted.

Nurses participating in this study expressed an overall appreciation for working in a hospital with single bedrooms, but challenges were identified in terms of layout, patient safety, and the use of technology. By promoting an environment that sustains these factors, hospitals have the potential to greatly enhance both patient safety and the effectiveness and well-being of nurses. Hence, exploring deeper how technological advancements can complement the layout of hospitals with single bedrooms would be beneficial. Additionally, further exploration of the traditional role of nurses within such environments is necessary to gain a deeper understanding.

## Figures and Tables

**Table 1:** Participants’ characteristics.

Interview Number	Age	Department	Specialization in Nursing	Years of Nursing Experience
1	42	Surgical	No	19
2	41	Medical	No	16
3	25	Medical	No	3
4	51	Surgical	No	21
5	53	Surgical	No	30
6	28	Medical	No	3
7	32	Medical	Yes	8
8	31	Surgical	No	7
9	48	Medical	No	11
10	32	Medical	No	10
11	33	Medical	No	11
12	34	Medical	No	13

**Table 2:** An excerpt from the analyses of the subcategory “the encumbrance of technology”.

Important Sentences and Words	Initial Reflections	Codes
“It is not enough to be a good nurse in this hospital. There are so many systems that you have to master in order to work here”	Nurses have to reshape their role and adapt to new systems	Mastering systems, change of role
“I believe that much more time is spent on administration, documentation and looking at the computer than before”	Time spent on computers have increased	Increased time on computer usage
“It’s just not enough equipment for us…we have external workstations, we have two external workstations and we also have three heavy-duty workstations. We have lack of space. We don’t have anywhere to work, we dont have enough computers which is unfortunately but those are our working tools. So it needs to be adapted a bit more to accomodate us”	Nurses workstations and available computers are not adapted to their needs	Limitations in equipment and workspace
“So, oh my God, the computer systems are great… but the network is so slow, you get kicked out. One of the systems jumps from one patient to another, it’s not quality assured…no, there are definitely some discrepancies, I think. You have to stay on top of i, yes... and we don’t have enough computers...ideally, it would have been to have a computer in every room actually, I think. And it needs to go a bit faster, the system is way to slow”	The nurse acknowledge the different systems, but express frustrations about lack of computers and the performance of the network	Lack of space and equipment, systems not functioning
“There are many different systems and there’s a lot that nees to be documented in very many different places. It takes time. So, we have a lot of checklits, like remember to document here and there…”	When the same thing must be documented in different systems, it's time consuming and nurses have checklists to remember to document in different systems	Multiple places to document is time consuming
“It takes a lot of time to log into the computers with all the technical problems and errors and all that, and you have to click through and find everything”	Nurse encounter that it is time consuming to log in and experience technical problems	Log in problems
“We had to document in four different places, so that we would get the correct score”	Nurses have to document in multiple places in order to score patients correctly	Multiple places to document
“The electronic medication system is better because there is no risk of misinterpreting the doctors’s writing, so you don’t have to decipher the handwriting, you know… that’s good becuase it gives you a clearer overview. SO, I would say the electronic medication system is an improvement compared to the previous paperbased system, but we are still waiting for it to get even better because the network is so slow”	Nurse state that the electronic medication system minimizes misinterpretertation of doctors' handwriting, but that the network i slow	Medication administration, quality improvement, slow system
“We get degraded when the doctors are coming, and then we are left to use the portable computers. We have to stand an document out in the corridors”	Nurses feel degraded because they have to give up their workstations to the doctors and they have to perform their tasks in the corridor	Sense of being degraded because lack of space
“If you must sit and read for an hour to get to know the patients, there is something wrong, You have to see to the patients. You have to use your eyes”	Nurse believe that getting to know patients should ideally come from direct interaction and observation rather than extensive reading on the computer	Time consumption, computers, change of role
“I’m proficicent with the technology and have received necessary training. Overall, it's been manageable. I have a good grasp of the systems. The only issue is that they can be unwieldy or operate slowly, which can be timeconsuming”	The nurse is positive about her own capability with the technology and have received sufficient training, but think that the technical systems can operate slow	Competence, sufficient training, system performance is slow

**Table 3:** An excerpt from the analyses of the subcategory “The enhanced communication”.

Important Sentences and Words	Initial Reflections	Codes
“If it’s questions about life and death, or drug problems at home, children… it is much easier to address these things and talk openly about them.”	Sensitive matters can be talked about more freely in a single bedroom	Sensitive matters
“The patients can say, nah…I have already informed my co-patient about this, so I don’t mind.”	Patients sometimes share information with co-patients	Sharing information with others
“It is significantly easier to communicate with patients in a single room, it really is.”	Single bedroom offers better communication with patients	Enhanced communication
“Yes, it is easier, there is no need to consider that there is someone else there. You can... yeah, you can be more free... if it’s someone you can joke around with, while another patient might be more anxious and you can’t use as much humor.. or if there are sensitive pieces of information that come up, or... let’s say topics that one wants to discuss.”	The nurse finds it easier to adjust to individual differences in communication styles when the patient is alone and it creates a more comfortable environment for conversations	Adjusting to the patients’ needs
“Yes... when the patients are in single rooms, then it’s easier for us to talk a bit more freely with them, in a way. There’s no one in the other bed who can listen”	It is easier to communicate with the patient without the concern of being overheard	Talking freely when no one listens
“I often feel like they open up a bit more.”	Patients open up more to the nurses when in a single bedroom	Opening up to the nurses
“But also, perhaps at least some patients, if you ask a few intimate questions, as one often tends to do before the doctor’s visit and so on, you notice that they find it a bit uncomfortable to respond to that when they are in a double room versus a single room.”	Patients can feel uneasy when answering intimate questions in a double room versus a single bedroom	Answering intimate questions in front of others can be uncomfortable
“It depends on the individual. Whether they are open or not, whether they feel it’s okay to talk like that in front of others. Everyone has their own struggles, and I think you don’t open up as well when there’s someone in the neighboring bed who can hear everything that is said, in a way... Because you don’t know if the person in the other will be telling things further, and even if they don’t know the patients and their medical history, they still pick up on it.”	Communication in a hospital setting can be difficult when addressing personal and sensitive matters, especially if there are more patients in the room that can listen in on what is being said. Sharing a room can raise privacy concerns and fear of one’s medical story being shared	Fear of eavesdropping
“It’s the confidentiality that... that is maintained... which you don’t have the opportunity for in multi-bedrooms.”	Confidentiality is maintained in a single bedroom	Confidentiality in single bedrooms
“I was just about to say that. The thing is, that the conversation is improved, both from our side and from the patient’s side. They can say things they wouldn’t have said if there had been another patient there. We have some two-bed rooms in... this ward, which due to not wanting to have so many corridor patients, we have converted the largest isolation rooms into two-bed rooms, which we have to use when there is overcrowding, and they are almost always in use. Then it becomes a bit more complicated with communication, one might say.”	The nurse observed that the quality of communication between healthcare providers and patients is enhanced when in a single bedroom where patients feel secure and at ease enough to share their thoughts.	Single bedrooms make it easier and safer to communicate

**Table 4:** Overview of the categories and the subcategories.

Being Present	Being Absent
The enhanced communication	The lonely patient
The placement of patients	The encumbrance of technology
The quality of nursing	Time-consuming layout

## Data Availability

The data are not publicly available due to confidentiality.

## Public Involvement Statement

No public involvement in any aspect of this research.

## Guidelines and Standards Statement

This manuscript was drafted against the CoreQ checklist https://www.equator-network.org/, accessed on 7 September 2023.
